# Hidden Penis

**Published:** 1875-01

**Authors:** 


Penis Hidden by the Scrotum.—Dr. C. O. Wright reported the case of a child one year old, in which the penis was almost entirely enveloped in the scrotum, only the glans protruding. The penis could be traced beneath the scrotum, which was movable. Asked whether it would be advisable to resort to an operation for the purpose of freeing the organ, or to leave it as it is.

Dr. Conner said the practice in such cases is to operate, dissecting out the penis, and either covering the organ with a flap, or allowing it to granulate over. Such operations were usually successful.—*The Clinic.*

Epitaph on Sir Fielding Ould, M.D., an Eminent Midwifery Practitioner in Dublin in the Last Century:—

"Sir Fielding Ould was made a Knight;
He should have been a Lord by right;
For then each lady's prayer would be,
O Lord! good Lord! deliver me."

